# Massive Intracerebral Hemorrhage Following Injudicious Use of Combined Oral Contraceptive Pills

**DOI:** 10.7759/cureus.35078

**Published:** 2023-02-16

**Authors:** Vidya Gaikwad, Sanjay Ponde, Suneha Yalla, Suhas Gaikwad

**Affiliations:** 1 Obstetrics and Gynecology, Dr. D. Y. Patil Medical College, Hospital & Research Centre, Dr. D. Y. Patil Vidyapeeth, Pune (Deemed to be University), Pune, IND

**Keywords:** ocp, uterine fibroid, craniectomy, haemorrhagic stroke, intracerebral haemorrhage, oral contraceptive pills

## Abstract

Contraceptive methods have taken various shapes since their inception and the most widely used are oral contraceptive pills (OCPs). In addition to contraception, OCPs have a variety of uses in the treatment of a number of gynaecological disorders, including polycystic ovary syndrome (PCOS), endometriosis, irregular menses, menorrhagia and dysmenorrhea. Since they were first introduced, OCPs have been linked to a higher risk of intracranial haemorrhage (ICH) and stroke. We report a case where the patient irrationally took OCPs for a long period of time and presented to the emergency department in a state of altered sensorium with symptoms of vomiting and headache which are suggestive of hemorrhagic stroke.

## Introduction

Over 100 million women use oral contraceptive pills (OCPs) as a reliable family planning method globally. According to a recent study, 8% of married women take OCPs [1]. In 1961 [2], the first account of a lady experiencing stroke while taking OCPs was published. In addition to being used for contraception, OCPs are used in the treatment of gynaecological disorders like polycystic ovary syndrome (PCOS), endometriosis, irregular menses, menorrhagia and dysmenorrhea [3]. Since the introduction of OCPs, their use has been linked to a higher risk of venous and arterial thrombosis [4]. Women with genetically inherited or acquired risk factors for thrombosis had higher rates of venous and arterial thrombosis [5]. Due to their oestrogenic nature, OCPs appear to be a significant contributor to cerebral venous sinus thrombosis (CVST) as oestrogen raises coagulation factor levels while lowering anticoagulant protein levels [6]. Young women are less likely to experience strokes than older women. The baseline incidence of stroke in women under 35 is estimated to be six to 20 per 100,000 and the incidence rises with age. About one-third of strokes result in death, and their long-term effects can be severe [7]. Here, we report the case of a patient who irrationally consumed OCPs for a long period of time and presented to the emergency with clinical symptoms of hemorrhagic stroke such as altered sensorium, headache and vomiting.

## Case presentation

**Figure 1:**
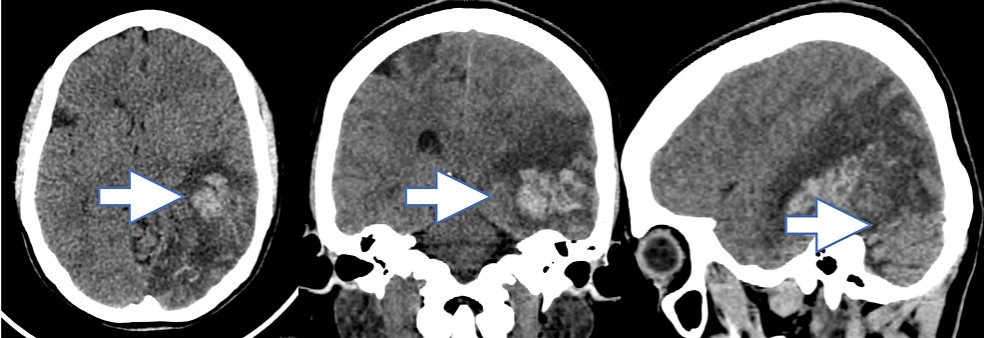
CT Brain showing intraparenchymal hemorrhage in parietal (axial), temporal (coronal) and occipetal (sagittal) views.

A 40-year-old third para presented to the emergency room in a state of altered sensorium with complaints of vomiting and headache for one day. She complained of heavy menstrual bleeding for one year. CT Brain showed an intraparenchymal haemorrhage in the left temporo-parieto-occipital (junction is located at the posterior end of the Sylvian fissure, where the temporal, parietal and occipital lobes meet) region of 3.8*2.7*2.2 cm (Figure 1). There was no family history of venous thromboembolism. Her body mass index (BMI) was 31. She was prescribed high dose combined OCPs (Ovral G: norgestrel 0.5 mg, ethinyloestradiol 0.05 mg) for the control of heavy menstrual bleeding (HMB), by a general practitioner. She irrationally took these pills at a dose of two pills, twice daily for seven days, multiple times, over a period of five months. She underwent left frontotemporoparietal decompression craniectomy with left temporal haematoma evacuation from the superior gyrus. She received four units of packed cells and four units of fresh frozen plasma in the postoperative period. One week later the prothrombin time and international normalized ratio (INR) of the patient were 12.7 and 1.1 respectively. She received injections of low molecular weight heparin for one month. Her pelvic ultrasound suggested an intramural fibroid of size 4*4 cm. She continued tablet norethisterone 20 mg, in divided doses with adjustment of the dose of warfarin, according to prothrombin time (PT)-INR levels for six months.

After six months, pelvic ultrasound showed a bulky uterus of size 9.6*6.9*7.3 cm with anterior wall intramural fibroid of size 4.7*4 cm with focal adenomyosis. She underwent a total abdominal hysterectomy with bilateral salpingectomy (TAH with BSO) after stabilisation of her PT-INR level. Her postoperative recovery was uneventful. Patient was reviewed by medicine, neurology and neurosurgery postoperatively. She was discharged eight days after hysterectomy and was advised to follow up for cranioplasty after two months.

## Discussion

Stroke, a cerebrovascular disease has a significant mortality and morbidity rate [8]. The American Heart Association's (AHA) 2018 update on heart disease and stroke statistics has reported that females in the United States have a greater lifetime incidence and mortality of stroke than men [9]. Vessey et al. reported for the first time that OCPs were linked to venous thromboembolism (VTE) and cerebral thrombosis in women. Numerous observational studies have since been carried out to evaluate the relationship between the usage of OCPs and the risk of stroke [10].

OCP use among women in developing nations is estimated to be 18% [11]. Tests of coagulation can be altered by contraceptive pills [12]. High doses of oestrogen and other risk factors such as smoking, hypertension and migraine history enhance the risk of ischemic stroke among OCP users. Additionally, the risk is increased in obesity, strong positive family history of stroke, prior OCP use and a history of venous thromboembolism [13].

Ovral G contains progesterone (norgestrel 0.5 mg) and oestrogen (ethinyl estradiol 0.05 mg) and is also known as combined oral contraceptive (COC). Over a five-month period, our patient misused it numerous times. The current usage of OCPs may slightly raise the risk of hemorrhagic stroke, however increased risk is linked to subarachnoid rather than intracerebral hemorrhage [14]. In our case, the patient misused high-dose OCPs, causing derangement of her coagulation profile ultimately resulting in massive intracerebral hemorrhage.

In the most recent Cochrane analysis, all combined oral contraceptives analysed were linked to a higher risk of venous thrombosis. Both the progestogen and the ethinylestradiol dosage affected the extent of impact. The risk of venous thrombosis with COCs containing 30-35 μg of ethinylestradiol with progestins-gestodene, desogestrel, cyproterone acetate and drospirenone was similar and about 50‐80% higher than with levonorgestrel. It is recommended to prescribe a combined oral contraceptive with levonorgestrel and 30 μg of ethinylestradiol, which has the lowest possible ethinylestradiol content and high compliance [15]. The increased risk of total, ischaemic and hemorrhagic stroke were all substantially correlated with higher oestrogen dosages. While its effects on hemorrhagic stroke risk were minimal, the longer duration of OCP usage significantly elevated the risk of total and ischaemic stroke. 

Therefore, the case discussed, in coherence with previous studies, concurs with the dose-dependent relationship between OCP use and the risk of stroke. It also provides a reference for using OCPs and preventing of cerebrovascular illnesses. Further research is required to investigate the potential underlying causes of OCPs' elevated brain stroke risk [16].

## Conclusions

The injudicious use of OCPs can lead to many unprecedented complications such as thromboembolism and stroke. These fatal outcomes can be avoided by taking a few simple precautions. Proper risk assessment and counselling are mandatory prior to starting any woman on OCPs. It is our responsibility to prescribe the correct COCs, regarding the appropriate dose, duration of intake and advice regarding timely follow up which will ultimately prevent life-threatening complications such as massive haemorrhagic stroke.
